# Sleep disturbances and stress among foreign medical students at European University, Georgia

**DOI:** 10.15694/mep.2019.000235.1

**Published:** 2019-12-17

**Authors:** Bedrye Soakin, Nidhi Maharaj, Irine Sakhelashvili

**Affiliations:** 1 European University

**Keywords:** Sleep, stress, foreign medical student, somatic pre-sleep arousal, cognitive pre-sleep arousal.

## Abstract

This article was migrated. The article was marked as recommended.

**Introduction**: The present study is the first attempt to assess the stressors associated with students’ life, daytime sleepiness, and pre-sleep arousal and to study the relationship between stress and sleep disturbance among foreign medical students in Georgia.

**Methodology**: Forty-four foreign volunteer students from the European University, Tbilisi, Georgia participated in this study in November 2018. Participants completed Epworth the Sleepiness Scale (ESS), Pre-Sleep Arousal Scale (PSAS), and Student-Life Stress Inventory (SLSI), and the data were statistically analysed.

**Results**: ESS scores were slightly higher than the normal value in the general population (7.25 ± 3.00). Students revealed high somatic (13.55 ± 4.39) and cognitive PSAS scores (18.05 ± 6.13) and high overall self-rating (1.95 ± 0.57) and total SLSI scores (143.86 ± 40.33). ESS scores were slightly higher in females (7.86 ± 3.00 vs 6.64 ± 2.94). Females showed higher somatic (13.73 ± 3.71 vs 13.36 ± 5.07) and cognitive PSAS scores (18.36 ± 5.34 vs 17.73 ± 6.94). Overall, self-rating and total SLSI scores were slightly higher among males (2.00 ± 0.69 vs 1.91 ± 0.43 and 146.55 ± 42.48 vs 141.18 ± 38.86, respectively). The differences were not statistically significant.

In females, only the cognitive pre-sleep arousal was significantly correlated with ESS (p<0.05), overall self-rating (p<0.05), and total SLSI scores (p<0.05).

In males, ESS showed significant correlation with both somatic and cognitive PSAS scores (p<0.05). ESS showed significant association with overall self-rating (p<0.01), which in turn was significantly associated with somatic and cognitive arousal (p<0.05). Both somatic and cognitive arousal were strongly correlated with total SLSI scores (p<0.01).

**Conclusion**: Medical students are at high risk of developing sleep disturbance and psycho-behavioural difficulties. Additional studies are needed to assess the specific relationship among sleep patterns, stress, and academic performance of foreign medical students.

## Introduction

A vast amount of scientific literature has documented the high prevalence of stress among university students worldwide. A previous study reported that students in United States showed a high prevalence of stress ( Regehr, Glancy and Pitts, 2013). In Pakistan, 57% of the medical students reported high levels of stress ( Waqas *et al.,* 2015). Stress was reported in 42% of Malaysian students ( Sherina, Rampal and Kaneson, 2004), while In Thailand, 61% of students reported stress ( Saipanish, 2003). Stress-inducing factors or the so-called stressors may include increased responsibilities, lack of coping skills, and deficient adaptation to a new environment. These stressors are linked to neurological and psychological reactions ( Ramli *at al.*, 2014). Students from the medical faculty are especially vulnerable to the development of stress and different psychological disturbances owing to dense curricula, tough schedules, and financial issues (Yusoff, Abdul Rahim and Yaacob, 2010). In case of foreign students, the stressors are intensified owing to cultural incompetence, language differences, stereotypes, and other complications.

Stress negatively affects almost every domain of student health and well-being. High stress levels were associated with decreased cognitive functioning, affected academic performance, and caused sleep disturbance ( Ahrberg *et al*., 2012). It is suggested that psycho-physiological factors such as stress, anxiety, and hyperactivity often play a major role in sleep disturbance. Stress is considered one of the most common precipitants of sleep disorders. A significant association was found between negative appraisal of stressors and sleep difficulties ( Morin, Rodrigue and Ivers, 2003). Moreover, studies have shown that somatic and cognitive arousals before bedtime play an important role in sleep quality and might cause poor sleep. Insomnia and other types of sleep disturbance are strongly associated with missing study hours, impaired memory, and increased risk of developing psychological difficulties ( Jansson-Fröjmark and Norell-Clarke, 2012). It is well established that a sufficient amount of sleep is necessary for recovering different body functions, memory consolidation, and the learning process ( Alvarez and Ayas, 2004; Killgore, 2010). Thus, sleep-deprived students may fare poorly with respect to marks and academic achievements compared to their peers. To our knowledge, only a few studies have evaluated the nature and the prevalence of the sleep problems among foreign medical students.

More than 80% of nearly 12,000 foreign students studying in Georgia are MD students. ( National Statistics Office of Georgia, 2019). Despite the growing number of foreign students, their health and psychological well-being has not been studied yet. We believe that the present study is the first attempt to evaluate stress levels, sleep characteristics, and pre-sleep arousal among foreign medical students in Georgia.

### The study objectives were to examine

1. The prevalence of daytime sleepiness, somatic and cognitive pre-sleep arousal, and different stressors among foreign medical students at the European University, Tbilisi, Georgia.

2. The differences in the prevalence of sleep difficulties and stress levels between male and female students.

3. The correlations among sleep disturbance, pre-sleep arousal, and stress in foreign medical students.

## Methods

### Study Participants

In November 2018, 44 foreign medical students (mean age 21.62 ± 1.80 years) studying at the European University, Tbilisi, Georgia participated in the study. The study was approved by the local University Ethical Committee in accordance with the principles of the Declaration of Helsinki. All the students in the study were volunteers. Signed informed consent was obtained from all the participants. There were no ethnic or gender preferences or other restrictions. The data collected in this study were obtained specifically for research purposes.

### Measures

The Epworth Sleepiness Scale (ESS) ( Johns, 1991) was used to measure daytime sleepiness. It is a self-administered questionnaire that assesses the overall level of daytime sleepiness of a subject. It is a measure of the probability of falling asleep in different situations and comprises of 8 questions. The respondent rates her/his chances of having taken a nap or having fallen asleep on a 4-point scale (0-3) while being involved in 8 widely different activities.

The total ESS score (a sum of scores across the 8 items) demonstrates the appraisal of a general characteristic, namely a person’s tendency to fall asleep across a wide range of activities in everyday life.

The Pre-sleep Arousal Scale (PSAS) ( Nicassio *et al.,* 1985) is a 16-item questionnaire designed to analyse the symptoms of cognitive and somatic arousals experienced at bedtime. Ratings range from 1 (not at all) to 5 (extremely). Severity of the somatic pre-sleep arousal is calculated by the summing the scores across the first 8 items. The sum of the scores across the last 8 items denotes the severity of the cognitive pre-sleep arousal.

The Student-Life Stress Inventory (SLSI) ( Gadzella, 1994) was used in the present study to assess lifetime stressors in the participants. The inventory consists of 51 items distributed across 9 sections, which indicate various types of stressors (frustration, conflict, pressure, changes, and self-imposed stressors) and reactions to stressors (physiological, emotional, behavioural, and cognitive) perceived by the participants. In the last question (question 52), the subject assesses her/his overall stress level using the Likert scale from a score of 1 (mild) to 3 (severe).

Demographic information and data on life attributes such as age and gender were collected from all the subjects.

### Statistical analysis

Stress, sleep variables, and demographic information (age and gender) were described using numbers and percentages for the categorical variables and mean ±standard deviation for the continuous variables. Independent-samples t-tests were performed to evaluate the differences in the mean values of the variables between the groups. The relationship between sleep and other variables was investigated using the Spearman’s correlation or the Pearson’s correlation. The confidence interval was set at 95%. Statistical analyses were performed using IBM SPSS Statistics 21.0 (IBM Corp, Armonk, NY, USA).

## Results/Analysis

The incidence of sleep difficulties and the severity of stress in students differed between the gender groups. The daytime sleepiness scores were slightly higher than the normal value in the general study population (7.25 ± 3.00). Moreover, the students revealed high levels of both somatic (13.55 ± 4.39) and cognitive pre-sleep arousals (18.05 ± 6.13). The overall self-rating SLSI score (1.95 ± 0.57) and the total SLSI score (143.86 ± 40.33) were above the normal range. We also performed comparisons between the male group and the female group to examine the gender differences among the variables. Excessive daytime sleepiness scores were slightly higher in female students (7.86 ± 3.00 vs 6.64 ± 2.94). The female students also showed considerably higher levels of both somatic (13.73 ± 3.71 vs 13.36 ± 5.07) and cognitive pre-sleep arousals (18.36 ± 5.34 vs 17.73 ± 6.91). The overall self-rating SLSI scores and the total SLSI scores were slightly higher in male students (2.00 ± 0.69 vs 1.91 ± 0.43 and 146.55 + 42.48 vs 141.18 ± 38.86, respectively). However, the differences were not statistically significant.

Descriptive statistics of the main variables are presented in Table 1.

**Table 1.** Descriptive statistics of general population

Variables (Mean ± SD)	Male N=22 (50%)	Female N=22 (50%)	Total n=44	p-value
Age	22.51 ± 1.41	20.72 ± 1.71	21.62 ± 1.80	**.001**
ESS	6.64 ± 2.94	7.86 ± 3.00	7.25 ± 3.00	.177
Somatic PSAS	13.36 ± 5.07	13.73 ± 3.71	13.55 ± 4.39	.785
Cognitive PSAS	17.73 ± 6.91	18.36 ± 5.34	18.05 ± 6.13	.735
SLSI overall self-rating	2.00 ± 0.69	1.91 ± 0.43	1.95 ± 0.57	.602
SLSI total score	146.55 ± 42.48	141.18 ± 38.86	143.86 ± 40.33	.664

*Note: SD, Standard Deviation; ESS, Excessive Daytime Sleepiness; PSAS, Pre-Sleep Arousal; SLSI, Student- Life Stress Inventory*

Compared to female participants, male participants showed higher stress levels in most of the SLSI dimensions, but the difference was statistically significant only for the scores of experienced pressure (p<0.01). However, the difference between the students’ frustration scores was almost significant (p=0.056). Results of the comparison are shown in Table 2.

**Table 2.** Gender Differences between the dimensions of Student-Life Stress Inventory

Variables (Mean± SD)	Male (N=22)	Female (N=22)	p-value
frustrations	18.95 ± 5.71	15.68 ± 5.32	**.056**
Conflict	7.72 ± 2.57	6.77 ± 3.44	.303
Pressure	12.95 ± 3.75	9.82 ± 3.41	.006 ^**^
Changes	8.77 ± 3.18	8.32 ± 2.4	.595
self-imposed	18.36 ± 5.31	16.05 ± 5.21	.152
Reacphysio	28.05 ± 10.13	27.14 ± 5.94	.719
ReacEmot	10.59 ± 4.88	11.59 ± 4.08	.465
ReacBehav	16.09 ± 6.09	13.77 ± 4.2	.150
CognitApp	6.32 ± 2.55	5.82 ± 2.52	.517
Overall self-rating	2.0 ± 0.69	1.91 ± 0.43	.602
SLSI total score	146.55 ± 42.48	141.18 ± 38.86	.664

** Significant correlation at the 0.01 level (two-tailed)

* Significant correlation at the 0.05 level (two-tailed)

*Note: frustrations, frustration experienced as a student; Conflict, stress after conflict; Pressure, stress experienced due to pressures; Changes, stressors due to changes; self-imposed, stress due to personal feelings; Reacphysio, physiological reactions to stress; ReacEmot, Emotional reactions to stress; ReacBehav, Behavioural reactions to stress; CognitApp, Cognitive Appraisal of the reactions to the stress; Overall self-rating, self-rating of overall level of stress;* *SLSI, Student-Life Stress Inventory*

In the general study group, excessive daytime sleepiness scores revealed significant correlations with the cognitive PSAS scores (0.518; p<0.01) and with the overall self-rating and total SLSI scores (0.539; p<0.01 and 0.376; p<0.01, respectively). Somatic pre-sleep arousal was significantly associated with the total SLSI scores (0.520; p<0.05), while cognitive pre-sleep arousal was significantly associated with the overall self-rating and total SLSI scores (0.518; p<0.01 and 0.460; p<0.01, respectively). Correlations between the main variables of the general study group are presented in Table 3.

**Table 3.** Correlations between sleep and stress variables in general population

N=44		SomaticPSAS	CognitivePSAS	SLSI overallself-rating	SLSI total score
ESS	***r***	.256	.518 ^**^	.539 ^**^	.376 ^*^
	***p***	.093	**.000**	**.000**	**.012**
Somatic PSAS	***r***	1	.460 ^**^	.234	.520 ^**^
	***p***		**.002**	.127	**.000**
Cognitive PSAS	***r***	.460 ^**^	1	.441 ^**^	.544 ^**^
	***p***	**.002**		**.003**	**.000**
SLSI overall self-rating	***r***	.234	.441 ^**^	1	.563 ^**^
	***p***	.127	**.003**		**.000**
SLSI total Score	***r***	.520 ^**^	.544 ^**^	.563 ^**^	1
	***p***	**.000**	**.000**	**.000**	

** Significant correlation at the 0.01 level (two-tailed)

* Significant correlation at the 0.05 level (two-tailed)

*Note: ESS, Excessive Daytime Sleepiness; PSAS, Pre-Sleep Arousal; SLSI, Student-Life Stress Inventory*

In female students (N=22) excessive daytime sleepiness showed significant correlation with the cognitive PSAS (0.566; p<0.01). The somatic PSAS showed no association with any of the variables. The cognitive pre-sleep arousal showed significant correlation with ESS (0.566; p< 0.05), with overall self-rating SLSI scores (0.475; p<0.05), and with total SLSI scores (0.466; p<0.05). Results are presented in Table 4.

**Table 4.** Correlations between variables in female foreign participants

N=22 (Female)		SomaticPSAS	CognitivePSAS	SLSI overallself-rating	SLSI total score
ESS	***r***	-.029	.566 ^**^	.400	.400
	***p***	.897	**.006**	.065	.065
Somatic PSAS	***r***	1	.077	-.408	.382
	***p***		.732	.059	.079
Cognitive PSAS	***r***	.077	1	.475 ^*^	.466 ^*^
	***p***	.732		**.025**	**.029**
SLSI overall self-rating	***r***	-.408	.475 ^*^	1	.332
	***p***	.059	**.025**		.132
SLSI total score	***r***	.382	.466 ^*^	.332	1
	***p***	.079	**.029**	.132	

** Significant correlation at the 0.01 level (two-tailed)

* Significant correlation at the 0.05 level (two-tailed)

*Note: ESS, Excessive Daytime Sleepiness; PSAS, Pre-Sleep Arousal; SLSI, Student-Life Stress Inventory*

In male students (N=22), ESS was significantly correlated with both somatic and cognitive pre-sleep arousals (0.470; p<0.05 and 0.492; p<0.05, respectively). Even stronger correlation was found between ESS and the overall self-rating SLSI scores (0.705; p<0.01), which in turn were significantly correlated with both somatic and cognitive arousals (0.531; p<0.05 and 0.437; p<0.05, respectively). Both somatic and cognitive pre-sleep arousals were significantly correlated with the total SLSI scores (0.625; p<0.01 and 0.612; p<0.01, respectively). Results of the correlational analysis of male foreign students are shown in Table 5.

**Table 5.** Correlations between variables in male participants

N=22 (male)		SomaticPSAS	CognitivePSAS	SLSI overallself-rating	SLSI total score
ESS	***r***	.470 ^*^	.492 ^*^	.705 ^**^	.401
	***p***	**.027**	**.020**	**.000**	.064
Somatic PSAS	***r***	1	.674 ^**^	.531 ^*^	.625 ^**^
	***p***		**.001**	**.011**	**.002**
Cognitive PSAS	***r***	.674 ^**^	1	.437 ^*^	.612 ^**^
	***p***	**.001**		**.042**	**.002**
SLSI overall self-rating	***r***	.531 ^*^	.437 ^*^	1	.707 ^**^
	***p***	**.011**	**.042**		**.000**
SLSI total score	***r***	.625 ^**^	.612 ^**^	.707 ^**^	1
	***p***	**.002**	**.002**	**.000**	

** Significant correlation at the 0.01 level (two-tailed)

* Significant correlation at the 0.05 level (two-tailed)

*Note: ESS, Excessive Daytime Sleepiness; PSAS, Pre-Sleep Arousal; SLSI, Student- Life Stress Inventory*

## Discussion

Current knowledge has confirmed that medical students experience high levels of stress in higher educational institutions due the content-heavy curriculum. The enormous stress induced by the tough schedule is exaggerated by the adjustment required to a new environment in an unfamiliar country. According to some studies, clinical depression is more frequent among medical students compared to the general population ( Helmers *et al*., 1997).About one-third of the sample from the 333 highest-grade students in the Los Angeles Area reported high levels of daily stress ( Anda *et al*., 2000).Our results confirm the findings of the previous studies ( Dahlin, Joneborg and Runeson, 2005) that medical students experience constant pressure and different types of stress. As anticipated, all the participants in the present study reported their stress levels with moderate or high scores. Male foreign students were especially vulnerable to life stressors. Higher scores in favour of male students in our study are not consistent with evidence from other studies, which showed that female medical students suffered more from stress ( Eva *et al.,* 2015; Wahed and Hassan, 2017). Despite these conflicting results, the bigger picture from our study is similar to other studies, confirming that the stress level scores were above the normal range in the general group and in both the sub-groups based on gender.

The participants from our study also revealed different types of sleep disturbance. We found that the students were under the risk of developing sleep difficulties and psycho-behavioural disturbances. Our findings are in accordance with previous studies, which confirmed a high prevalence of sleep problems and different psychological difficulties in high school students (Wolf and Rosenstock, 2017; Friedrich and Schlarb, 2018). Pre-sleep arousals, particularly the cognitive pre-sleep arousals, are directly related to the risk of developing insomnia, anxiety, and/or other psycho-physiological disorders (Palagini *et al.,* 2018). Therefore, the results of the state of the foreign students before bedtime is essential and worth paying attention to. Similar to the daytime sleepiness scores, the average values of pre-sleep arousals, especially the cognitive pre-sleep arousals in our study exceeded the normal values, indicating the presence of augmented rumination, anxiety, and worries among the students. All the aforementioned abnormal manifestations might be triggered by increased stress and sleep deprivation and/or other sleep difficulties ( Yeh, Wung and Lin, 2015; Ellis *et al*., 2018).

The strong association of lifelong and academic stress with sleep problems, which in turn are associated with poor health and other psycho-physiological aspects, is already well known.

Nevertheless, the influence of a completely unfamiliar environment with a content-heavy curriculum on sleep disorders and psychological well-being of foreign medical students in Georgia has not yet been thoroughly studied and requires more attention.

It is necessary to evaluate the specific relationship among sleep patterns, stress, and academic performance of foreign medical students. The results of the present study, along with the available evidence, emphasise the need to support students, especially the foreign ones, in overcoming stress and related difficulties. Universities that focus on internationalised programs to attract international students should consider university-based mental health providers.

It is also important to identify effective interventions to promote healthy development and well-being of students, which enable them to meet the challenges of various everyday conditions and socio-cultural contexts.

### Advantages and limitations of the study

The main limitation of our study is the small number of participants, which makes it impossible to generalise the results. However, we believe that our study is the first to provide the data regarding stress levels and sleep disorders among foreign medical students in Georgia.

Despite the exponentially growing number of foreign students in our country in recent decades, the health and other psycho-physiological parameters of such students have not yet been studied. The present study is the first attempt to start bridging this gap. This project is ongoing and the results with big data will be presented in future.

Meanwhile, on a larger scale, scientifically accurate description of sleep disorders and associated risk factors is an important scientific challenge. The results of the present study could lay the foundation of a new avenue of research in the recognition, prevention, and treatment of sleep problems and stressors in Georgia.

## Conclusion

Medical students, especially the male foreign students are at high risk to develop sleep problems and psycho-behavioural disturbances, as supported by the evidence in the present study. However, more data needs to be collected and analysed for better understanding of the relationship between sleep problems and stress among foreign medical students. Further research with a large sample size is needed to determine the physiological and psychosocial factors affecting students’ sleep, cognitive performance, and consequently, their academic achievements.

## Take Home Messages

• The learning environment affects student health and well-being.
• Medical students are under constant pressure and experience several types of stress and sleep problems. Male students are particularly vulnerable to these problems.
• Foreign medical students are at a high risk of developing sleep problems and psycho-behavioural disorders.
• Universities implementing international programs should consider service providers to facilitate the adaptation of the foreign students to the unknown environment.
• Daily stress and sleep-related problems negatively affect the cognitive abilities of the students and may result in poor academic performance.

## Notes On Contributors

Bedrye Soakin is a student of the medical faculty of European University, Tbilisi, Georgia. She distributed information sheets, obtained informed consent, and performed data collection.

Nidhi Maharaj is a student of the medical faculty of European University, Tbilisi, Georgia. She distributed information sheets, obtained informed consent, and performed data collection.

Irine Sakhelashvili, MD, PhD is a Professor at European University, Tbilisi, Georgia. She designed and implemented the project and developed the manuscript for publication.

## Declarations

The author has declared the conflicts of interest below.

Prof. Irine Sakhelashvili is a MedEdPublish Reviewer Panel Member.

## Ethics Statement

This study was approved by Institutional Review Board of European University, Tbilisi, Georgia; Approval number: E/1-18.09.2018.

## External Funding

This article has not had any External Funding
