# Supplementary material for: Sleep disturbances and stress among foreign medical students at European University, Georgia
Source: MedEdPublish (2016). 2019 Dec 17;8:235. [Version 1] doi: 10.15694/mep.2019.000235.1 (PMC10712639; doi:10.15694/mep.2019.000235.1)
Supplement: Peer review history

# Reviewer report: Reviewer response for version 1

**DOI:** 10.21956/mep.19929.r30227

**Published:** 2020-03-16

**Authors:** Ken Masters

**Affiliations:** 1 Sultan Qaboos University

This review has been migrated. The reviewer awarded 4 stars out of 5 An interesting paper covering sleep disturbances and stress among foreign medical students. The paper begins by firmly establishing stress as a problem among medical students, and then sets out to study stress and its correlation to sleep disturbance and other sleep problems among foreign medical students at the university, using established tools.Overall, the study is well-conducted, and the results very clearly presented. The Discussion relates the results to our current knowledge from other literature.While the sample size is a little small for detailed statistical analysis, the paper is still a useful read. It would, however, be more valuable for readers if some other background information about the students as foreign students could be included, in addition to some discussion about the student support systems. In addition, if the study were to be repeated, qualitative comments from the students would provide deeper insight into some of the cause of the sleep problems, especially on the pre-sleep arousals.Although the authors do not make the error of causation, their Discussion (and the first sentence of the Conclusion) does tend to imply that the sleep problems are as the result of stress, and this is a little dangerous, as the causality may operate in the opposite direction.

Reviewer Expertise:

NA

No decision status is available

# Reviewer report: Reviewer response for version 1

**DOI:** 10.21956/mep.19929.r30226

**Published:** 2019-12-20

**Authors:** P Ravi Shankar

**Affiliations:** 1 American International Medical University

This review has been migrated. The reviewer awarded 4 stars out of 5 This is an interesting study about stress and sleep disturbances among foreign medical students at a university in Georgia. The authors mention about the problems of students in adapting to a foreign environment. Medical school is stressful and students often move away from their support systems to a different location and environment. More information about the medical school, the total number of students admitted per year, the percentage of foreign students and support mechanisms available to these students will be helpful. Is instruction offered in Georgian or in English? Are the foreign students offered courses to learn the language? A similar situation exists in offshore Caribbean medical schools. Some of these schools offer different support mechanisms to help students adapt to the school, the curriculum and life in a foreign country. There have been a number of studies on stress among university students, in general and health science students in particular and while it may not be possible to cite all these studies the authors could consider including a wider selection of studies from developing nations. The manuscript is well written and will be of interest to all medical educators. I am very happy that students are the first and second authors of the paper.

Reviewer Expertise:

NA

No decision status is available
